# Stimulation of medulloblastoma stem cells differentiation by a peptidomimetic targeting neuropilin-1

**DOI:** 10.18632/oncotarget.24521

**Published:** 2018-02-16

**Authors:** Caifeng Gong, Julie Valduga, Alicia Chateau, Mylène Richard, Nadia Pellegrini-Moïse, Muriel Barberi-Heyob, Pascal Chastagner, Cédric Boura

**Affiliations:** ^1^ Université de Lorraine, CNRS, CRAN, F-54000 Nancy, France; ^2^ Service d'Onco-Hématologie Pédiatrique, CHRU-Nancy, F-54000 Nancy, France; ^3^ Université de Lorraine, CNRS, L2CM, F-54000 Nancy, France

**Keywords:** medulloblastoma, neuropilin-1, cancer stem cells, peptidomimetic, cell differentiation

## Abstract

Medulloblastoma (MB) is the most common malignant pediatric brain tumor. Despite the progress of new treatments, the risk of recurrence, morbidity, and death remains important. The neuropilin-1 (NRP-1) receptor has recently been implicated in tumor progression of MB, which seems to play an important role in the phenotype of cancer stem cells. Targeting this receptor appears as an interesting strategy to promote MB stem cells differentiation. Cancer stem-like cells of 3 MB cell lines (DAOY, D283-Med and D341-Med), classified in the more pejorative molecular subgroups, were obtained by *in vitro* enrichment. These models were characterized by an increase of NRP-1 and cancer stem cell markers (CD15, CD133 and Sox2), meanwhile a decrease of the differentiated cell marker Neurofilament-M (NF-M) was observed. Our previous work investigated potential innovative peptidomimetics that specifically target NRP-1 and showed that MR438 had a good affinity for NRP-1. This small molecule decreased the self-renewal capacity of MB stem cells for the 3 cell lines and reduced the invasive ability of DAOY and D283 stem cells while NRP-1 expression and cancer stem cell markers decreased at the same time. Possible molecular mechanisms were explored and showed that the activation of PI3K/AKT and MAPK pathways significantly decreased for DAOY cells after treatment. Finally, our results highlighted that targeting NRP-1 with MR438 could be a potential new strategy to differentiate MB stem cells and could limit medulloblastoma progression.

## INTRODUCTION

Medulloblastoma (MB) is the most common malignant pediatric brain tumor and affects children at a median age of 9 years [1]. Despite the progress of radio- and chemotherapy, the risk of recurrence, frequent cognitive and endocrine sequelae and death after treatment remain important [2, 3]. Recent results showed that MB was composed of four molecular subgroups: WNT (Wingless), SHH (Sonic Hedgehog), Group 3 and Group 4, which correspond to different molecular and clinical characteristics. Indeed, patients of groups 3 and 4, also called non-WNT and non-SHH groups, frequently present metastasis and have a poor prognosis [4, 5]. MB is classified as an embryonic tumor in which brain tumor stem cells (BTSCs) are present in very low proportion. BTSCs can be characterized by expression of stem cell phenotypic markers such as CD133 or CD15 [6, 7] and has a peculiar interest in understanding the progression of MB [8]. This cell population generates tumors through the stem cell patterns of self-renewal and differentiation into multiple tumor cell types. Moreover, these cells have better DNA repair capability contributing to tumor resistance during radiation and chemotherapy. Conventional therapies could kill differentiated tumor cells, but the small population of BTSCs can survive and causes tumor recurrence [8–10].

Neuropilin-1 (NRP-1) is a single-pass transmembrane glycoprotein that plays a very important role in the development of neuronal and vascular systems, and shares about 44% homology with neuropilin-2 (NRP-2). It acts as a co-receptor by complexation with other transmembrane receptors such as VEGFR and plexin receptors, which are involved in neoangiogenesis and tumor progression by activating signaling pathways leading to cell survival, proliferation or migration [11]. Recently, Snuderl et al. demonstrated that PlGF acts through NRP-1 to promote MB cell survival but not through VEGFR1 [12]. Moreover, NRP-1 seems to favor an undifferentiated phenotype in cancer cells [13]. Targeting directly this receptor, especially in BTSCs, could thus provide interesting therapeutic value to change the fate of cancer stem cells to a differentiated state for improvement of survival and quality of life of medulloblastoma patients.

Our previous work focused on the development of peptides for targeting NRP-1 and we have proposed to use peptidomimetics for their theoretical stability [14, 15]. We have recently designed and assessed some new sugar-based peptidomimetics targeting NRP-1 and one of them named MR438 presented a relevant *in vitro* affinity for NRP-1 (IC_50_ of 88 μM) [16]. Tuftsin (TKPR: Thr-Lys-Pro-Arg) is a natural ligand of NRP-1 with a IC_50_ of 25 μM [17, 18] and it was used in our work as reference compound. Therefore, we investigated the exposition of these two compounds targeting NRP-1 on MB stem cells (obtained from 3 cell lines: DAOY, D283-Med and Med-D341) in order to assess their short-term effects as cytotoxicity and cell invasion or their long-term effects as self-renewing ability and the change of phenotypic status. We first characterized the 3 MB stem cell models which over-expressed NRP-1 and stem cell markers and found that inhibition of NRP1 decreased the self-renewing ability of MB stem cells by inducing their differentiation.

## RESULTS

### Phenotypic characteristics of MB stem cell models

**Figure 1:**
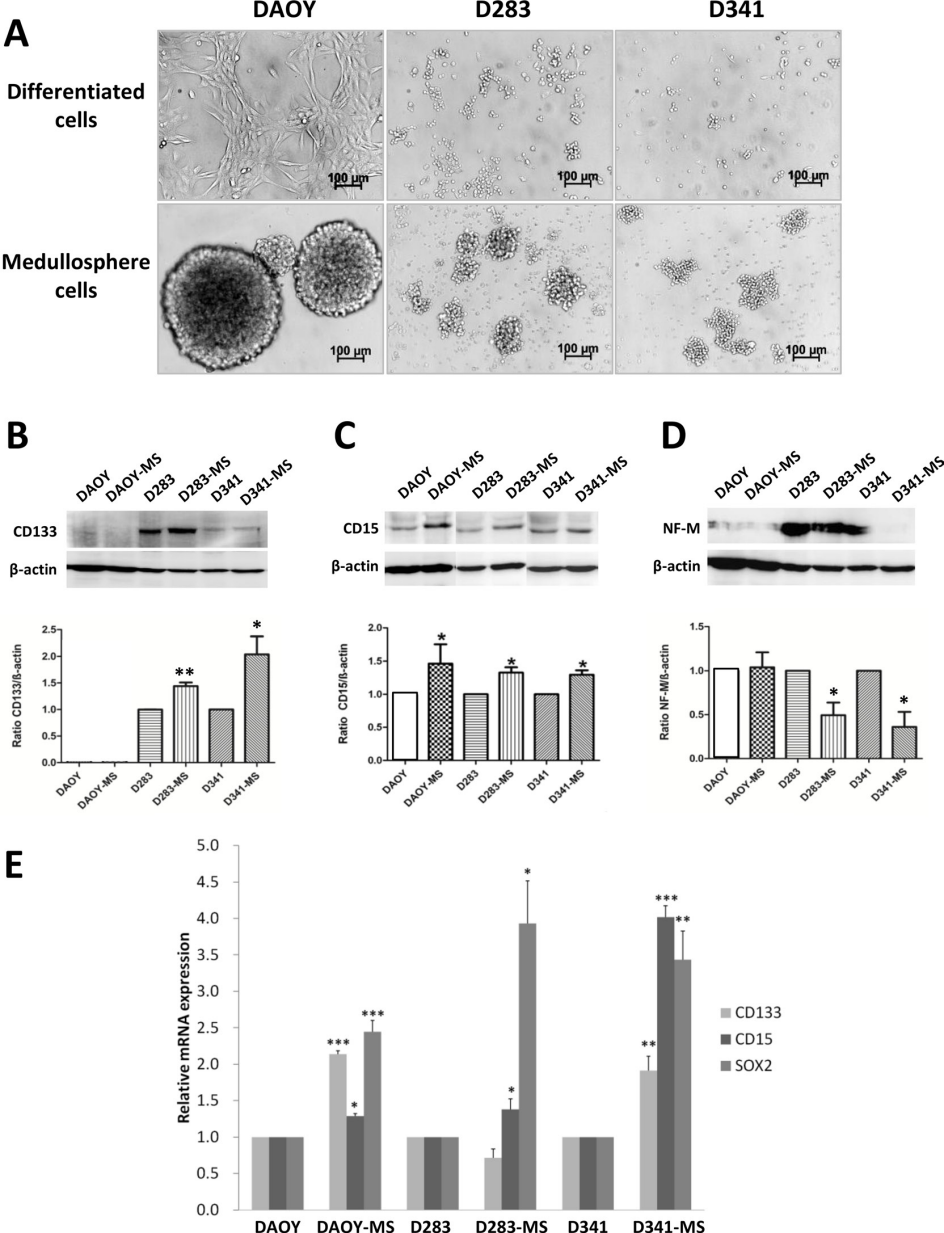
Phenotypic proteins and transcripts expression of MB stem cells models (**A**) Images of medullospheres of MB stem cells from cell lines: DAOY, D283-Med and D341-Med (× 40 magnification, Bars:100 μm). Expression of CD133 (**B**), CD15 (**C**) and NF-M (**D**) between differentiated cells and MB stem cells by Western blot normalized by β-actin expression. (**E**) Gene expression of phenotypic transcripts of CD133, CD15 and Sox2 of differentiated cells and MB stem cells normalized by RNA pol II expression. ^*^p < 0.05, ^**^p < 0.01, ^***^p < 0.001, *n* = 3.

Three cell lines of MB: DAOY, D283-Med and D341-Med were used *in vitro* to obtain medullospheres (MS) as MB stem cell models (Figure 1A). They correspond to the subgroup SHH, subgroup 4 and subgroup 3, respectively [5, 12, 19]. The medullospheres of DAOY were larger and more regular than the other two cell lines and reached a diameter of about 150 μm after a 72 h culture period. These models were characterized by protein expression of stem cell markers which showed, as expected, an increase in the expression of cancer stem cell markers: CD15 for all 3 models and CD133 for D283 and D341 compared to the differentiated cells (Figure 1B and 1C, Supplementary Table 1). A decrease of the neuronal differentiated phenotype marker, Neurofilament-M (NF-M), was also observed for the cells from medullospheres compared to the differentiated cells. Furthermore, because expressions of protein CD133 and NF-M for DAOY cells were very weak, we evaluated Sox2, another stem cell marker, which increased for the DAOY stem cells (Supplementary data, Supplementary Figure 1 and Table 2). These results confirmed by qRT-PCR and showed an increase of gene level expression of CD15 and Sox2 for all models of MB stem cell and of CD133 for DAOY and D341 compared to the differentiated cells (Figure 1D).

### Protein expression of neuropilins by MB stem cell models

**Figure 2:**
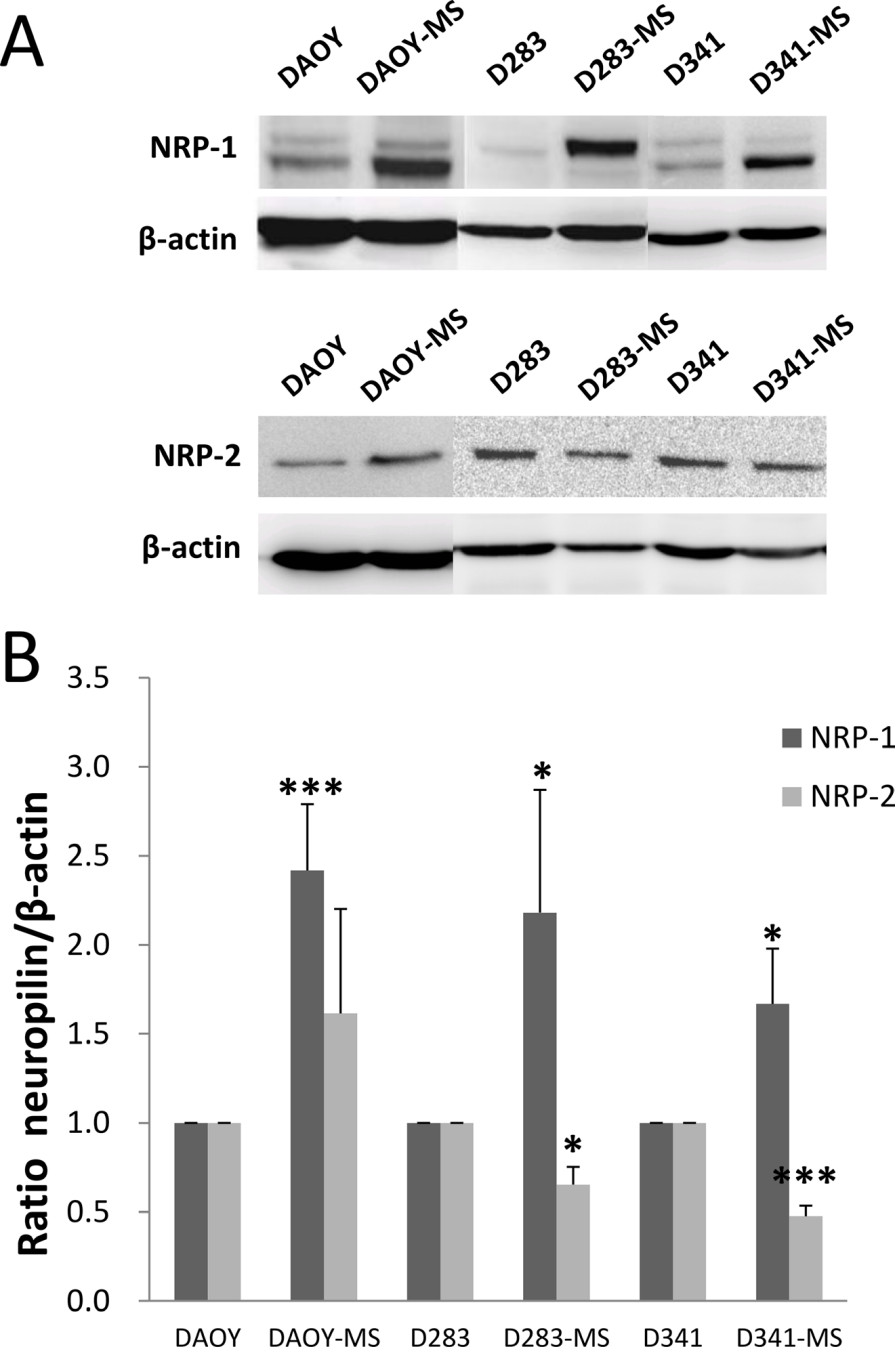
NRP-1 and NRP-2 proteins expression of MB stem cell models of DAOY, D283-Med and D341-Med by Western blot (**A**) Representative results of expression of NRP-1 and NRP-2 for differentiated cells and MB stem cells. (**B**) Ratio of NRP-1 and NRP-2 expression to β-actin protein for differentiated cells and MB stem cells. ^*^p < 0.05, ^***^p < 0.001, *n* = 4.

NRP-1 and NRP-2 play an important role in the development of neuronal and vascular systems. NRP-2 is a homologous protein that shares a sequence similarity of 44% in structural and biological properties with NRP-1 [20]. In our study, NRP-1 and NRP-2 were expressed by all cell lines of MB (Figure 2 and Supplementary Table 2). Meaningfully, there was a significant increase in the expression of NRP-1 protein (120 kDa) by MB stem cells compared to differentiated cells. A decrease of NRP-2 expression was observed for D283 and D341 stem cells compared to the differentiated cells.

### Effect of peptidomimetic MR438 on spheres formation and cell viability

**Figure 3:**
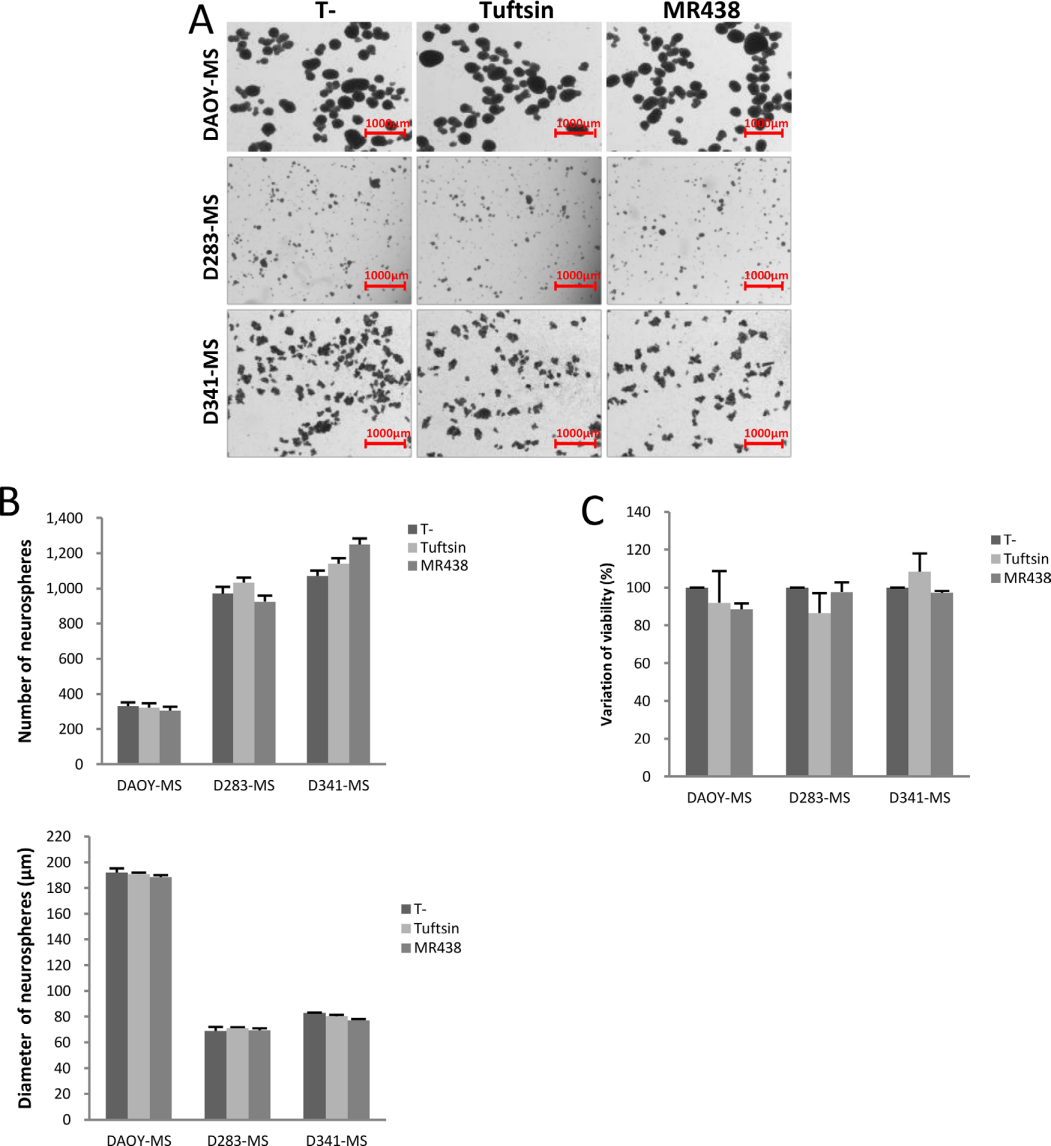
Effects of MR438 or Tuftsin on cytotoxicity and on spheres formation for MB stem cell models of DAOY, D283-Med and D341-Med (**A**) Representative images of medullospheres from the different cell lines exposed to MR438 and Tuftsin evaluating the sphere formation ability (Bars:1000 μm). (**B**) Effect of MR438 and Tuftsin on number and diameter of medullospheres. (**C**) Viability of cells contained in the medullospheres treated by MR438 and Tuftsin after 72 hours of exposition for three cell lines. MS: Medullosphere, *p* > 0.05, *n* = 6.

To detect the short-term effects of these compounds on medullospheres, we evaluated the ability of spheres formation (number and diameter of spheres) as well as the cell viability after 72 hours of treatment (Figure 3). DAOY rapidly formed numerous large spheres in serum free condition contrary to D283 and D341, but MR438 or Tuftsin were not able to influence the cell ability to form spheres for all cell lines (Figure 3A and 3B). In the same way, no significant difference was observed in cell viability for the different cell lines (DAOY-MS, D283-MS and D341- MS) (Figure 3C).

### Effect of peptidomimetic MR438 on self-renewal capacity

**Figure 4:**
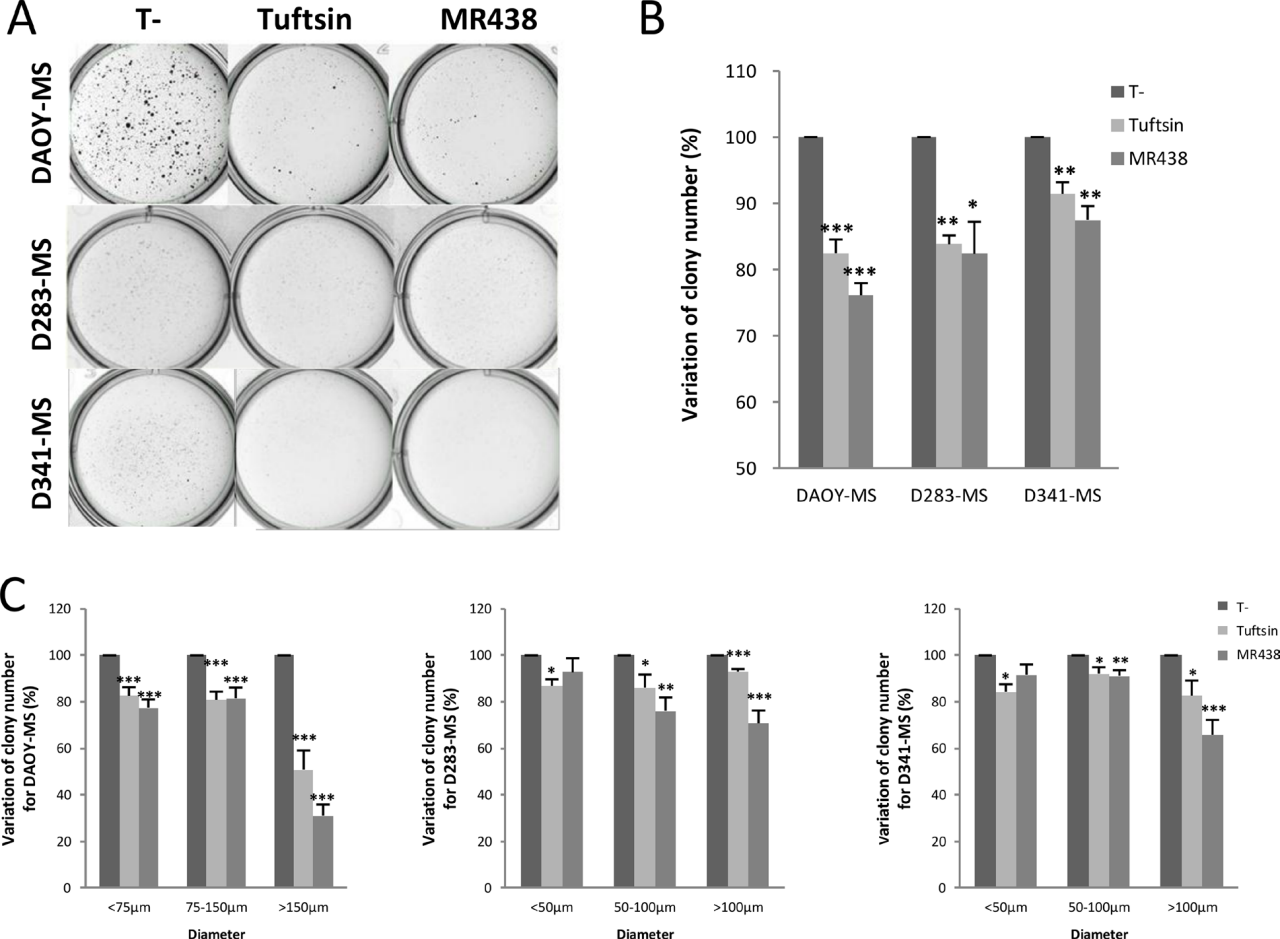
Effects of MR438 or Tuftsin on self-renewal ability by clonogenic assay for DAOY, D283 and D341 stem cells (**A**) Representative images of medullospheres treated with MR438 or Tuftsin by clonogenic assay using methylcellulose culture medium for three cell lines: 2^*^10^4^ cells/well for DAOY-MS and 5^*^10^4^ cells/well for D283-MS and D341-MS. (**B**) Ratio of colony number compared to control group. (**C**) Ratio of colony number compared to control group depending on different ranges of diameter for DAOY-MS, D283-MS and D341-MS. MS: Medullosphere, ^*^p < 0.05, ^**^p < 0.01, ^***^p < 0.001, *n* = 6.

Self-renewal is the process by which stem cells divide to make more stem cells, perpetuating the stem cell pool throughout life. Self-renewal is division with maintenance of the undifferentiated state. Self-renewal programs involve networks that balance proto-oncogenes (promoting self-renewal), gate-keeping tumor suppressors (limiting self-renewal), and care-taking tumor suppressors (maintaining genomic integrity). The effect of MR438 on self-renewal capacity was assessed by using forming colonies assay (Figure 4). The number of MB stem cells (colony forming units) was statistically significantly decreased after adding NRP-1 targeting compounds for 72 h, especially after exposition to MR438. We observed a reduction of about 25% of colonies of DAOY stem cells and approximately 20% of colonies of D283 and D341 stem cells. The colonies were divided into three groups according to their diameters: smaller than 75 μm (small clone), from 75 to 150 μm (normal clone) and higher than 150 μm (wide clone) for clones of DAOY-MS and less than 50 μm, 50–100 μm and more than 100 μm for colonies of D283-MS and D341-MS (Figure 4C). MR438 effects were mainly observed for wide colonies, especially for the DAOY cells, describing a mean reduction of 60% after MR438 exposition.

### Effect of peptidomimetic MR438 on expression of neuropilins and phenotypic markers by western blot and qRT-PCR

**Figure 5:**
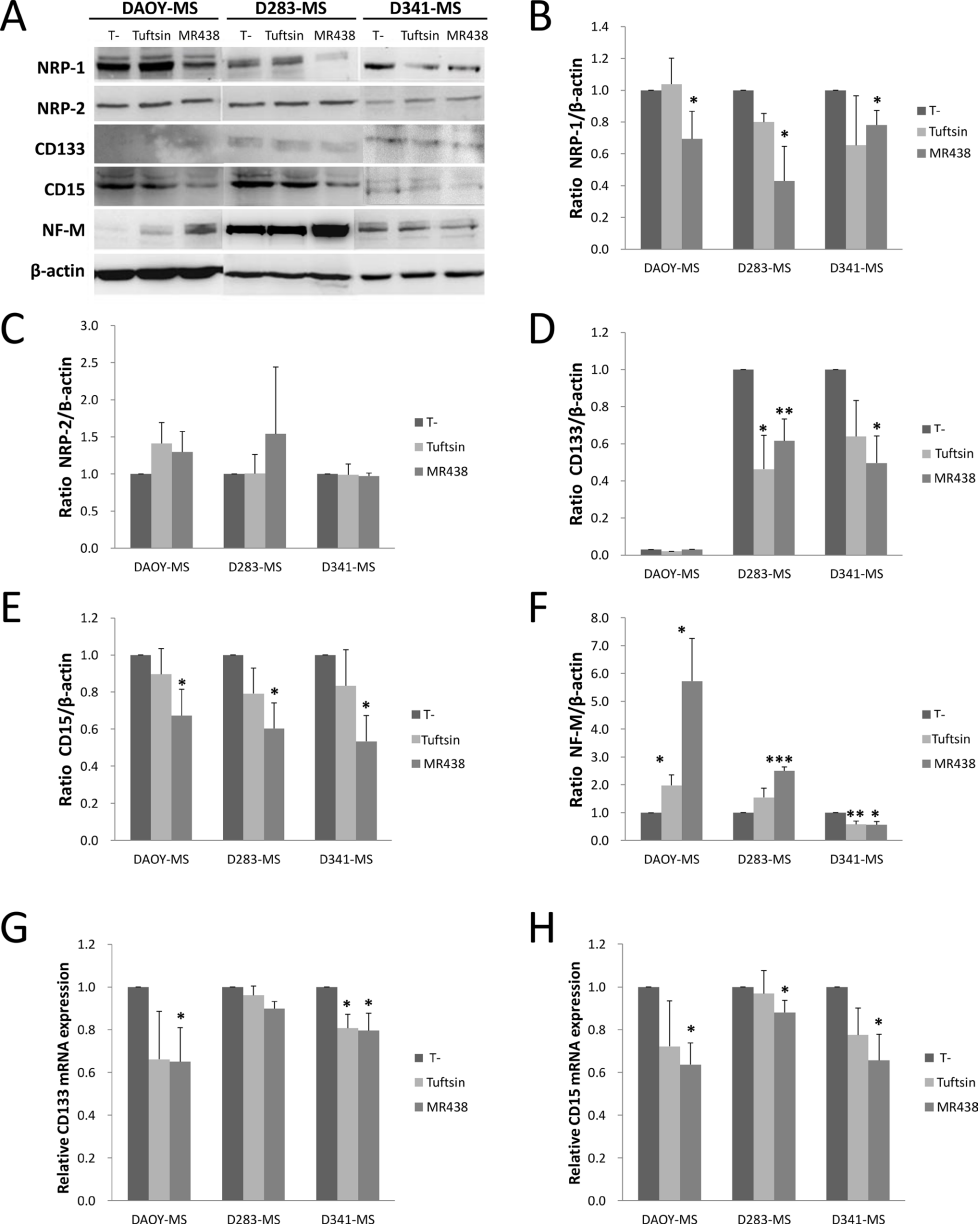
Effects of MR438 or Tuftsin on proteins and transcripts expression of neuropilin receptors and stem cell markers (**A**) Representative blots of expression of neuropilins and phenotype markers for MB stem cells exposed to MR438 or Tuftsin by western blot. Ratio of NRP-1 (**B**), NRP-2 (**C**), CD133 (**D**), CD15 (**E**) and NF-M (**F**) protein expression normalized by β-actin expression for MB stem cells treated by MR438 or Tuftsin. Effects of compounds on mRNA expression of CD15 (**G**) and CD133 (**H**) for MB stem cells by qRT-PCR. ^*^p < 0.05, ^**^p < 0.01, ^***^p < 0.001, *n* = 3.

After 72 h of expostion to MR438, a statistically significantly decrease of NRP-1 protein expression was observed for the 3 MB stem cell models Figure 5A and 5B, Supplementary Table 3 and confirmed also by flow cytometry, see Supplementary Figure 2). However, this compound did not modify the protein expression level of NRP-2 (Figure 5A and 5C, Supplementary Table 3). Otherwise, the influence of the compound on the expression of the phenotypic markers CD133 and CD15 and NF-M was measured by western blot analysis, demonstrating that MR438 reduced the expression of CD15 for the three cell lines (confirmed also by flow cytometry, see Supplementary Figure 2), and decreased the expression of CD133 for D283-MS and D341-MS ((Figure 5D and 5E, Supplementary Table 3). Since CD133 protein was undetectable for DAOY-MS of the SHH subgroup, we used another stem cell phenotypic marker Sox2 [21]. Sox2 expression decreased after exposure to MR438 by western blot (Supplementary Materials, Supplementary Figure 1 and Table 3). On the contrary, there was an increase in NF-M expression for DAOY-MS and D283-MS after exposure to MR438 (Figure 5A and 5F, Supplementary Table 3). These results were confirmed by qRT-PCR. We observed that the mRNA expression of CD15 and CD133 decreased significantly for the 3 cell lines after treatment with MR438 except for CD133 mRNA of D283-MS cell line (Figure 5G). Transcription factors Sox2, Oct4 and Nanog were also detected to supplement the impact of peptidomimetic on MB stem cells, whereas only a significant decrease of Sox2 for the D283-MS cell line was detected after the exposure to MR438 (Supplementary Materials, Supplementary Figure 3).

### Effect of peptidomimetic MR438 on invasive capacity of medullosphere cells

**Figure 6:**
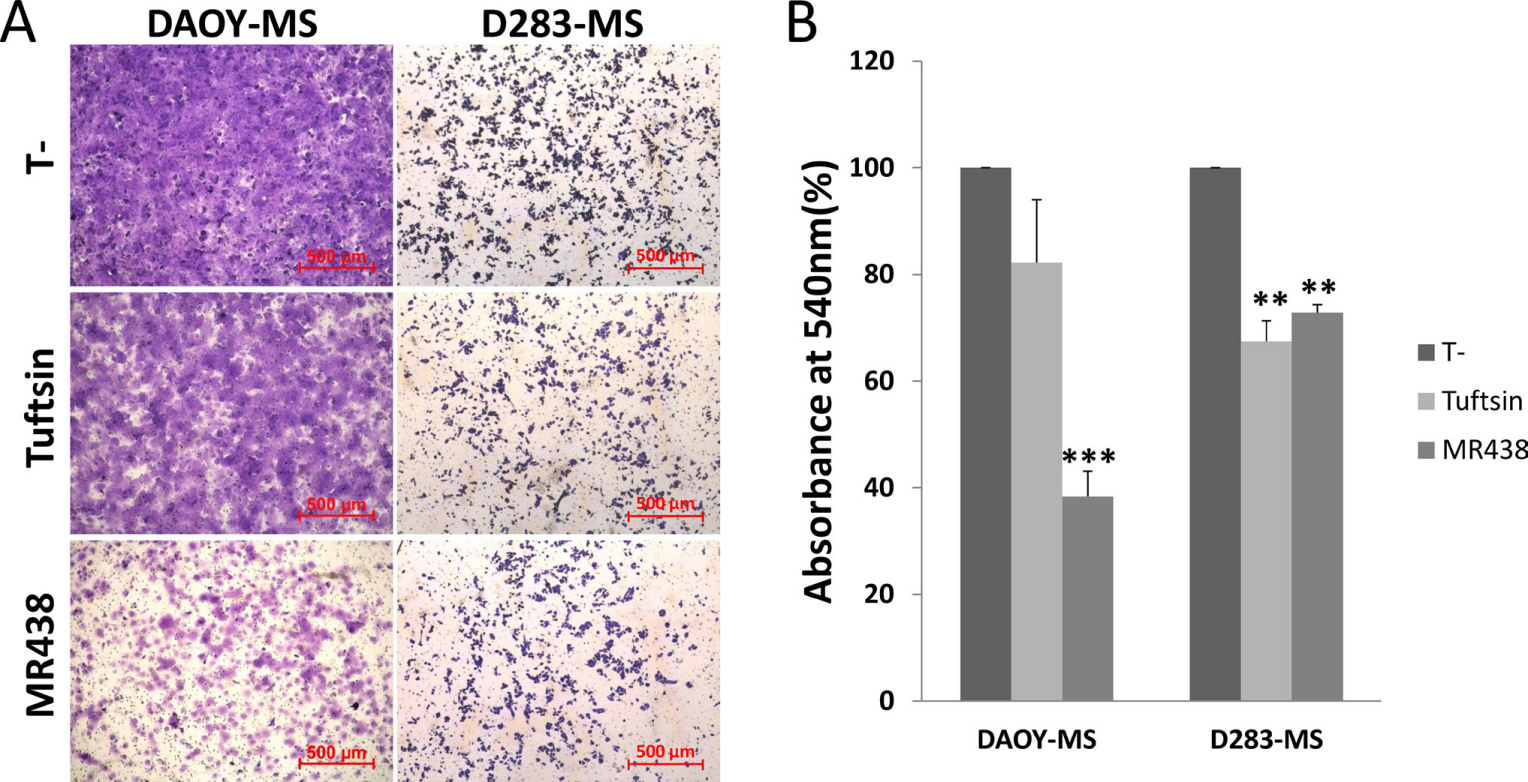
Effects of MR438 or Tuftsin on invasive ability of MB stem cells derived from DAOY and D283 cell lines (**A**) Images of invasive cells exposed to MR438 or Tuftsin on the membrane surface of Boyden chamber (Bars: 500 μm). (**B**) Ratio of invasive cells exposed to MR438 or Tuftsin compared with the no treated cells. ^**^p < 0.01, *n* = 3.

As NRP-1 is also involved in cell migration, we evaluated the effects of Tuftsin and MR438 on the invasive capacity of stem cells using the Boyden chamber model (Figure 6). MR438 induced a decrease in the capacity of invasion of MB stem cells derived from DAOY and D283 cell lines. Tuftsin is only able to inhibit invasion for D283 cell line. It was not possible to perform the invasion assay for D341 cells due to the limitations of these cells to adhere to Matrigel^®^ depiste the use of chemoattractant factors in the bottom chamber.

### Effect of peptidomimetic MR438 on the key proteins involved in the neuropilin pathways

**Figure 7:**
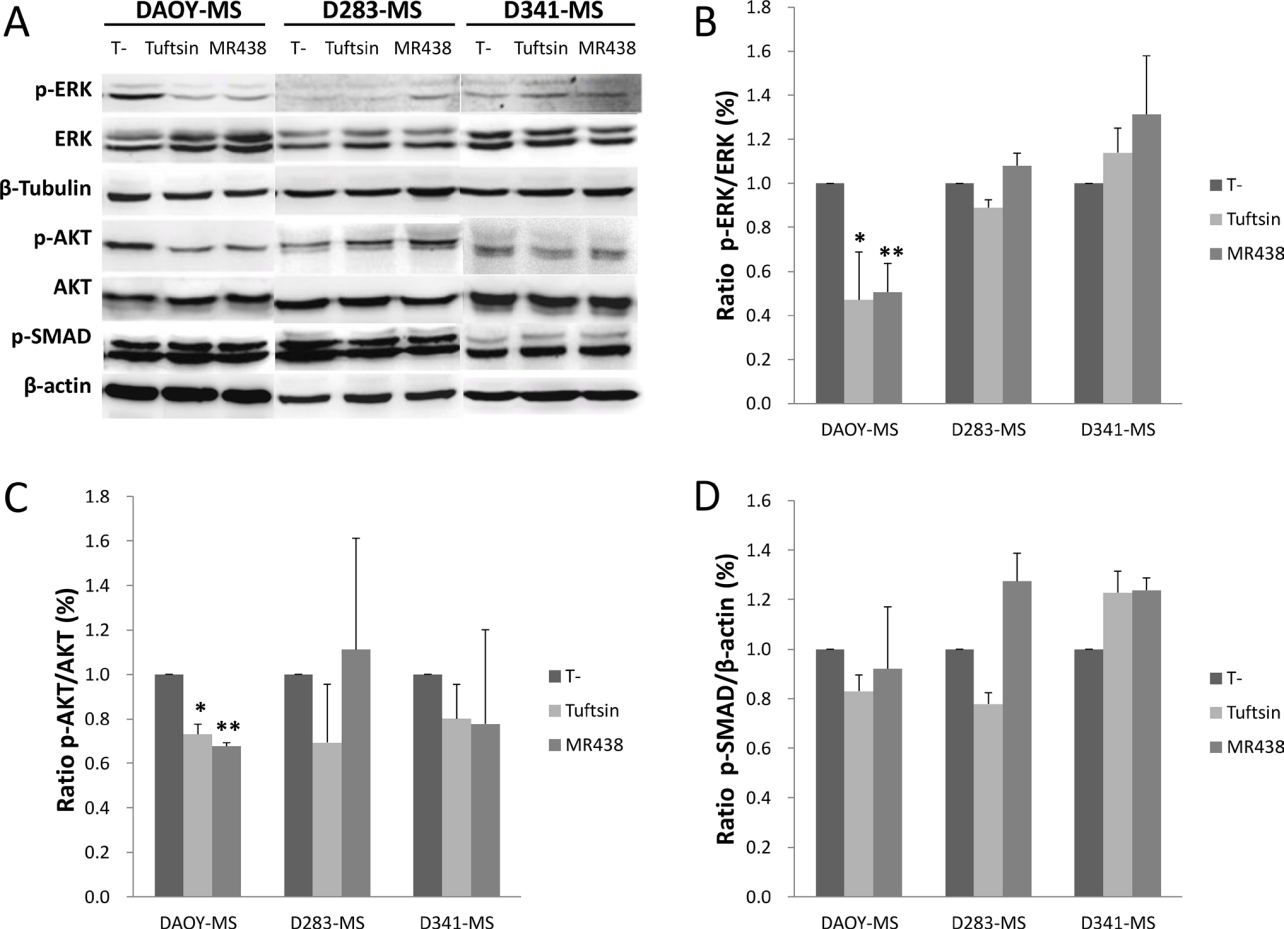
Effects of MR438 or Tuftsin on the key proteins involved in the neuropilin pathways (**A**) Representative blots of p-ERK, ERK, p-AKT, AKT, p-SAMAD2/3, β-actin and β-tublin by Western blot. (**B**) Ratio of p-ERK to ERK for MB stem cells treated by MR438 or Tuftsin. (**C**) Ratio of p-AKT to AKT for MB stem cells treated MR438 or Tuftsin. (**D**) Ratio of p-SMAD to β-actin protein for MB stem cells treated by MR438 or Tuftsin. ^*^p < 0.05, ^**^p < 0.01, *n* = 3.

The study of the main signaling pathways known to be regulated by NRP-1 was carried out by the analysis of the phosphorylation state of key proteins of PI3K/AKT, RAS/MAPK and SMAD signaling pathways (Figure 7 and Supplementary Table 4). According to the activation state of the RAS/MAPK pathway, the expression of phospho-ERK1/2 showed a significant decrease for DAOY-MS cells after treatment with MR438 and Tuftsin compared to the untreated cells (Figure 7A and 7B). However, this difference in phosphorylation was not found for the 2 other models, probably because of a low level of expression of this protein (Figure 7A). Similarly, phosphorylated AKT expression also appeared to be significantly decreased for DAOY-MS cells after exposure to MR438 and Tuftsin, but no difference was observed for the two other cell models (Figure 7A and 7C). As for the signaling pathway of AKT and ERK, there was no difference in the expression of phospho-SMAD2/3 after treatment with Tuftsin or MR438 whatever the cell models. There is a trend of increase of phospho-SMAD2/3 expression after treatment with Tuftsin or MR438 for D341 cells (Figure 7A and 7D).

## DISCUSSION

A better understanding of the molecular characteristics involved in MB allowed to subdivide this pediatric brain tumor into four molecular subgroups: WNT, SHH, Group 3 and Group 4, with different molecular characteristics and clinical outcomes and thus to consider new therapeutic approaches [4, 5]. The WNT and SHH groups were thus named in connection with signaling pathways which appear to play important roles in the pathogenesis of these subgroups. Subgroup 3 and 4 MB are related to a high incidence of metastasis and lead to a poorer prognosis. Thus, it is important to study as much as possible all the molecular subgroups of MB for a better understanding of this disease. Therefore, in our study, we analyzed the three most frequent used cell lines of MB: DAOY, D283-Med and D341-Med, corresponding to the subgroup SHH, subgroup 4 and subgroup 3, respectively [12, 19].

NRP-1 is a single-pass transmembrane glycoprotein, which acts as a co-receptor by complexing with many transmembrane receptors such as VEGFR and Semaphorin receptor, or with TGF-β1 receptor, as it has been recently demonstrated [20]. NRP-1 is involved in cell survival and proliferation and has been reported to be over-expressed in various cancers, which have also been correlated with poor prognosis [22–25]. More recently, Snuderl *et al.* found that MB patients with high NRP-1 expression level have a decrease of 50% in survival. Moreover, targeting placental growth factor (PlGF)/NRP1 with monoclonal antibodies induced a direct antitumor effect in MB, resulting in tumor regression, decrease of metastasis and increase of survival in mouse models [12]. In the context of a peptide based approach, Tuftsin, a natural peptide produced by enzymatic cleavage of the Fc domain of the immunoglobulin G heavy chain, was found to bind specifically to NRP-1 [18]. However, Tuftsin is not an ideal agent for use in clinical practice because of several limiting points including size, stability (susceptible to degradation by peptidases), lack of effective methods for delivery, low oral bioavailability, rapid excretion and poor transport properties through biologic membranes [26, 27]. Thus, we developed a novel peptidomimetic named MR438 which has been built by molecular modeling based on well-known A7R peptide [16, 28].

Recent studies report that cancer stem cells (CSCs) are considered to be the origin of tumor proliferation and involved in the tumor recurrence because of their resistance to radiotherapy and chemotherapy [29]. CD133 is the most commonly used stem cell marker used for the identification of brain cancer stem cells [6, 30], but it was also shown that CD15+ MB cells could recur and lead to poor prognosis in mouse models [7]. Indeed, CD15 is a carbohydrate antigen that is expressed on both progenitors and stem cells in the embryonic and adult central nervous system and was also recently considered as a marker of brain cancer stem cells especially for Sonic hedgehog (SHH) MB subgroup cells [7, 31]. As expected, our MB stem cells obtained in serum free conditions characterized by overexpression of stem cell markers, in particular CD15 for DAOY stem cells (SHH subgroup) and CD133 for D283 cells (subgroup 4) compared to cell lines cultured under classic conditions. Interestingly, the *in vitro* enrichment of cancer stem cells induced a NRP-1 overexpression for all cell lines, which is encouraging to consider NRP-1 targeting as a strategy applicable to different MB subgroups.

MBs are poorly differentiated tumors containing a large proportion of cancer stem cells, so acting on their differentiation could also play an assisting role in the therapeutic management of MBs [32]. Cao *et al.* found that NRP-1 helps in maintaining an undifferentiated phenotype in renal cell carcinoma [13]. In our study, we have not observed early effects of NRP-1 targeting on cell viability nor on the ability of cells to form medullospheres. However, we observed a decrease in self-renewal capacity in the presence of MR438 and to a lesser extent in presence of Tuftsin indicating that cancer stem cells probably enter in a differentiation way. This seems more efficient for SHH sub-group cells.

The dissemination of MB stem cells in hemato-meningeal space is a real clinical problem leading to metastasis occurrence and tumor recurrence [33]. Cao *et al.* showed also that the knockdown of NRP-1 by short hairpin RNA reduced migration and invasion of renal carcinoma cells [13] which was also found in our results with a decrease of invasion ability of MB stem cells after exposure to MR438. In the same way, it was recently shown that MiR-148a, a MiRNA which decreased NRP-1 expression, also inhibited invasion and tumorigenic potential of MB cells in the WNT subgroup [34].

Subsequently, we have endeavored to understand which signaling pathways of NRP-1 are preferably involved in the differentiation of MB stem cells. The precise signaling pathways of NRP-1 action are still unclear as they interact with many cancer associated molecules. It has been demonstrated that overexpression of NRP-1 in cancer cells promotes tumor angiogenesis and stimulates cancer stem cell feature that depends on the complex NRP-1/VEGFR-2 for the CD133(+) human glioma stem-like cells (GSCs) [35, 36]. VEGFR and other VEGFR co-receptors of NRP-1 are involved in neoangiogenesis, tumor progression and differentiation by activating signaling pathways like PI3K/AKT, MAPK and SMAD. Thus, the most marked effect of MR438 as well as Tuftsin was the inactivation of PI3K/AKT and MAPK pathways for the SHH subgroup cells (DAOY). Frasson *et al.* have also recently proposed that the most important pathway involved in cell differentiation of MB stem cells is the PI3K/AKT/mTOR pathway [37]. Although TGF-beta-dependent SMAD signaling pathway did not appear to be disrupted in our study, Nissen *et al.* reported that Tuftsin promotes Smad3 phosphorylation and reduces AKT phosphorylation by TGF-beta-dependent signaling pathway [38]. In addition, we did not observe the effect of MR438 on the expression of SHH, although the expression of NRP-1 is known to activate the SHH signaling pathway [39]. Other unknown functions of NRP-1 could explain the decrease in self-renewal capacity observed for the subgroups 3 and 4 and will have to be elucidated by unsupervised transcriptomic approaches.

In conclusion, by using models of MB stem cells, we found that inhibition of NRP-1 via the peptidomimetic MR438 seems to stimulate stem cell differentiation for different subgroups of MB, which can ultimately reduce the progression of MB, with an implication of the PI3K/AKT and MAPK signaling pathways for subgroup SHH. The use of NRP-1 targeting molecules seems relevant to target MB stem cells, notably by promoting their differentiation. Further molecular studies could help us understand the involved mechanisms and we will confirm these results in the future in *in vivo* medulloblastoma xenograft models.

## MATERIALS AND METHODS

### Cell culture of MB stem like cells

**Figure 8:**
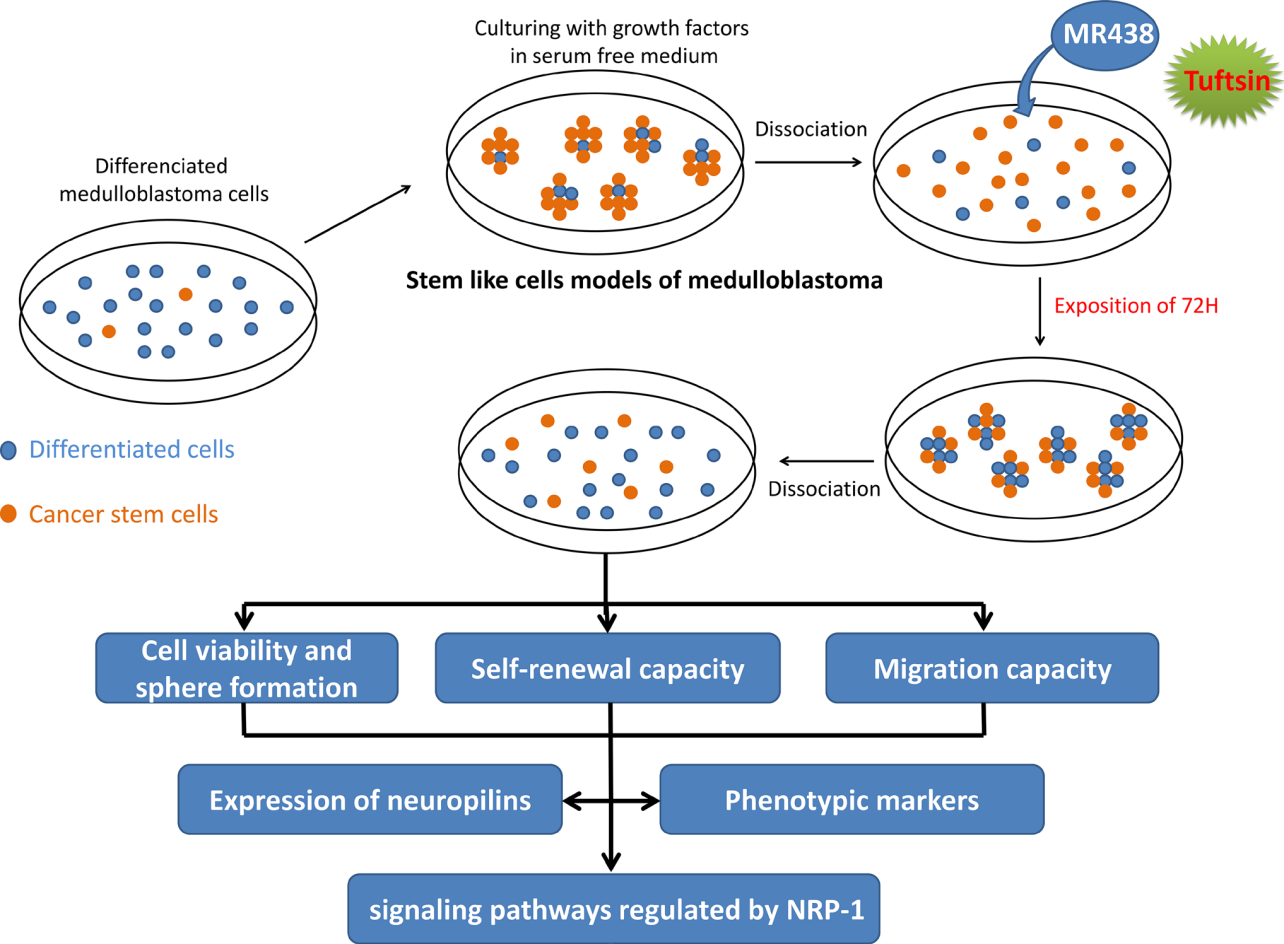
Design of the experimental procedures to evaluate the peptidomemitic effects on MB stem cells models obtained by *in vitro* enrichment methods

The human MB cell line DAOY [40], D283-Med [41] and D341-Med [42] were purchased from ATCC cell biology collection (Manassas VA, USA). Cells were maintained in MEM (Gibco, Life Technologies Corporation, UK) including 10% fetal bovine serum (FBS, SIGMA, USA) for DAOY and D283, 20% FBS for D341-Med, 1% L-glutamine (SIGMA, UK), 1% non-essential amino acids (Gibco, UK), 1% penicillin/streptomycin (Gibco, UK) and 1% Sodium pyruvate (Gibco, UK) at 37°C and 5% CO2. MB stem like cells cultures were maintained in DMEM/F12 medium (Gibco, UK) containing B27 and N2 supplement (Gibco, Life Technologies Corporation, USA), 20 ng/mL of human recombinant epidermal growth factor (EGF) and basic fibroblast growth factor (bFGF) (EGF and FGF from Miltenyi Biotec, Germany). After a 3-day culture in hydrophobic flasks at 37°C with 5% CO2 in a humidifier atmosphere, spheres were obtained. MB stem like cells were dissociated from spheres using Accumax (Gibco, Life Technologies Corporation, UK) and seeded in 75 cm^2^ or 25 cm^2^ flasks depending on the experiment. The MB dissociated stem like cells were then exposed to MR438 (Molecular weight: 527.20 g/mol, supplied by the SRSMC laboratory-UMR 7565 in powder form) and Tuftsin (Molecular weight: 500.60 g/mol, BACHEM, Switzerland) at 25 μmol/L during 72 h (Figure 8).

### Sphere formation and cells viability

We observed the sphere formation of the MB cells after a 72 h incubation with the compounds Tuftsin and MR438 at the concentration of 25 μM. The cells were seeded in 6 wells plates at a density of 60 000 cells/mL for DAOY-MS and D341-MS and 75 000 cells/mL for D283-MS. The number of neurospheres larger than 30 μm was quantified using GelCount™ (Oxford Optronix, UK) to count the number of spheres and to evaluate the efficacy of medullosphere formation. Each experiment was repeated 3 times with 3 independent wells.

The viability of MB stem like cells was evaluated by automated cell counter TC20 (Biorad, France) using the trypan blue exclusion assay in 24 well plates at the same condition of cell density like sphere formation assay. Spheres were previously dissociated with Accumax and cell suspension then rapidly stained with the same volume of 0.4% trypan-blue solution and deposited in counting chamber slides (TC20, Biorad). The percentage of surviving cells was counted twice and experiment was repeated 6 times.

### Clonogenic assay

After exposure of MB stem like cells to MR438 and Tuftsin, clonogenic growth assay using methylcellulose based media was done. Briefly, MB stem like cells (20000 cells/well for DAOY-MS and 50000 cells/well for D283-MS and D341-MS) were suspended in 2 ml of DMEM/F12 containing 1% methylcellulose (SIGMA, USA). The cell suspension was then plated onto 6 well plates and allowed to grow for 8–12 days. Colonies were incubated with 0.5% MTT solution (Thiazolyl blue tetrazolium bromide, 98%, Acros Organics™) and colonies larger than 30 μm in diameter were quantified using GelCount™ (Oxford Optronix, UK). Each experiment was repeated 6 times with 3 independent wells.

### Transwell invasion assays

Transwell invasion assays were performed using a transwell insert (Corning Incorporated, Corning, USA) with 4.4% Matrigel^®^ (BD Biosciences, France) coated onto the transwell membrane (8 μm pore size, 6.5 mm diameter). The lower chambers of the transwell plates were filed with 700 μL DMEM/F12 containing 10% FBS. Cells were cultured in the higher chambers (200 000 cells/chambre for DAOY-MS and 1000 000 cells/chambre for D283-MS) onto 24-well plate with 500 μL serum free DMEM/F12. After 16 h for DAOY-MS and 48 h for D283-MS in incubator at 37°C, cells on the upper surface of filters were removed using cotton swabs and those on the lower surface were fixed 10 minutes with 4% paraformaldehyde and staining with 0.05% crystal violet of 30 minutes. Photomicrographs of whole culture surface were taken (Nikon AZ100, Digital Sight DS-Qi1Mc camera, Nikon, France). The coloration was solubilized 10 minutes by placing the inserts in 150 μL of 4% acetic acid and then transferred into 96 well plates, the absorbance was read at 540 nm with a spectrophotometer (Thermo Electron Corporation, Finland).

### Analysis of proteins expression by western blot

Western blot was carried out for analysis of NRP-1, NRP-2, CD133, CD15, NF-M and Sox2 protein expression. Total protein cell lysis buffer containing 10% protease (Roche, Germany), 1% Cocktail 2 and 3 (Sigma-Aldrich, Germany) was used to lysis of cells to extraction proteins. Kit Pierce BCA Protein Assay (Thermo scientific, USA) was used to determine protein concentration that read the absorbance at 540 nm with a spectrophotometer. Protein aliquots (50 μg) were denatured in the Laemmli buffer containing Δ mercaptoethanol prior to resolution by SDS polyacrylamide gel electrophoresis. The separated proteins were transferred onto PVDF membranes (Biorad, USA). After blocking the PVDF membrane with Tris base-buffered saline prepared with 0.1% Tween-20 containing 5% bovine serum albumin within 1 hour, the following primary antibodies against NRP-1 (#3725, Cell Signaling, 1:1000 dilution), NRP-2 (#32241, Novus Biologicals, 1:1000 dilution), CD133 (#130-090-422, Miltenyi Biotec, 1:500 dilution), CD15 (#14-0159, eBioscience, 1:1000 dilution), Sox2 (#SAB5500176, SIGMA, 1:1000 dilution), NF-M (#2838, Cell Signaling, 1:1000 dilution), p-ERK (#9106, Cell Signaling, 1:2000 dilution), ERK (#9102, Cell Signaling, 1:1000 dilution), p-AKT (#9271, Cell Signaling, 1:1000 dilution), AKT (#9272, Cell Signaling, 1:1000 dilution), p-SMAD (#8828, Cell Signaling, 1:1000 dilution), β-tublin (#2128, Cell Signaling, 1:1000 dilution) and Δ- actin (#4970, Cell Signaling, 1:1000 dilution) were incubated overnight at 4°C. Quantification of relative band densities was performed using densitometer (LAS Imager FujiFilm) and Δ-actin or β-tublin was used as internal control.

### Gene expression of phenotypic markers by qRT-PCR

To confirm the protein expression, the gene expression of Sox2, Oct4, Nanog, CD133 and CD15 (Table 1) for the differentiated cells and stem cells in medullospheres, as well as the impact of MR438 and Tuftsin, were analyzed by quantitative reverse-transcription PCR (qRT-PCR). Total RNA was extracted with All Prep-DNARNA-Mini Kit (Omega). Reverse transcribed to cDNA was synthesized using the iScript™ cDNA synthesis Kit (BioRad) with conditions of 25°C for 5 minutes, 42°C for 30 minutes, 85°C for 5 minutes, and 12°C forever. Quantitative PCR amplification was performed with SyberGreen PCR supermix (BioRad) using the CFX96 Real-Time System (BioRad). The qPCR conditions were 95°C for 2 minutes and 39 cycles of 95°C for 5 seconds and 63~68°C for 30 seconds, the hybridization temperature was depended on primers (Table 1). All values were normalized to RNA pol II and the ΔΔCt method was used to estimate the fold change expression over control samples.

**Table 1:** Sequences and annealing temperatures of primers used in qRT-PCR

Gene	Primer sequence (5′–3′)	Tm (°C)
CD133 Fwd	TCCGGGTTTTGGATACACCCTA	68
CD133 Rev	CTGCAGGTGAAGAGTGCCGTAA	
CD15 Fwd	AGGAGGTGATGTGGACAGCG	67
CD15 Rev	AACTACGAGCGCTTTGTGCC	
Sox2 Fwd	TTTCACGTTTGCAACTGTCC	63
Sox2 Rev	AGTCTCCAAGCGACGAAAAA	
Oct4A Fwd	ACCTGGAGTTTGTGCCAGGGTT	68
Oct4A Rev	CTCCCCTGCCCCCACCCTTT	
Nanog Fwd	GATGGGAGGAGGGGAGAGGA	68
Nanog Rev	TTTGGAAGCTGCTGGGGAAG	
RNA pol II Fwd	TGGGCAAAAGAGTGGACTTC	64
RNA pol II Rev	TTGAAGGGGGTGACAATCTC	

Abbreviations: Fwd: forward, Rev: reverse, Tm: melting temperature, pol: polymerase.

### Statistical analysis

All results were given as mean ± standard error of the mean (SEM). Nonparametric test was employed to determine the statistical significance using SPSS Statistics 5 (SPSS Statistics 19.0, USA) with a minimum of 3 repetitions. For all figures, *p* < 0.05 (marked by ^*^) was considered significant and *p* < 0.01 and *p* < 0.001 were marked by ^**^ and ^***^, respectively.

## SUPPLEMENTARY MATERIALS FIGURES AND TABLES


