# Supplementary material for: Stimulation of medulloblastoma stem cells differentiation by a peptidomimetic targeting neuropilin-1
Source: Oncotarget. 2018 Feb 16;9(20):15312–25. doi: 10.18632/oncotarget.24521 (PMC5880606; doi:10.18632/oncotarget.24521)
Supplement: Supplementary file 1 [file oncotarget-09-15312-s001.pdf]

Stimulation of medulloblastoma stem cells differentiation by a peptidomimetic targeting neuropilin-1

SUPPLEMENTARY MATERIALS

Supplementary Table 1: Western blot densitometries of proteins CD133, CD15 and NF-M expression for differentiated cells and MB stem cells

Protein	CD133			CD15			NF-M		
Repition	1	2	3	1	2	3	1	2	3
DAOY	-	-	-	1.000	1.000	1.000	1.000	1.000	1.000
DAOY-MS	-	-	-	1.193	1.461	2.046	0.790	1.367	0.962
D283	1.000	1.000	1.000	1.000	1.000	1.000	1.000	1.000	1.000
D283-MS	1.325	1.426	1.567	1.189	1.324	1.469	0.702	0.561	0.220
D341	1.000	1.000	1.000	1.000	1.000	1.000	1.000	1.000	1.000
D341-MS	1.957	1.500	2.659	1.186	1.285	1.416	0.024	0.478	0.580

"-": no detectable, *n* = 3.

Supplementary Table 2: Western blot densitometries of proteins NRP-1, NRP2 and Sox2 expression for differentiated cells and MB stem cells

Protein	NRP-1			NRP-2			Sox2			
Repition	1	2	3	1	2	3	1	2	3	4
DAOY	1.000	1.000	1.000	1.000	1.000	1.000	1.000	1.000	1.000	1.000
DAOY-MS	1.863	2.815	1.918	2.311	0.446	2.085	2.904	1.549	1.791	7.458
D283	1.000	1.000	1.000	1.000	1.000	1.000	-	-	-	-
D283-MS	3.542	1.687	1.312	0.604	0.508	0.847	-	-	-	-
D341	1.000	1.000	1.000	1.000	1.000	1.000	-	-	-	-
D341-MS	2.275	1.483	1.245	0.641	0.410	0.476	-	-	-	-

"-": no detectable, $n \geq 3$.

Supplementary Table 3: Western blot densitometries of the effect of peptidomimetic on expression of neuropilins and phenotype markers for MB stem cells of MB

Protein		NRP-1			NRP-2			Sox2		
Repition		1	2	3	1	2	3	1	2	3
	T-	1.000	1.000	1.000	1.000	1.000	1.000	1.000	1.000	1.000
DAOY-MS	tufstine	0.943	1.170	1.074	0.866	1.583	1.791	0.852	0.908	1.038
	MR438	0.631	0.610	0.832	0.757	1.682	1.449	0.582	0.628	0.730
	T-	1.000	1.000	1.000	1.000	1.000	1.000	-	-	-
D283-MS	tufstine	0.592	0.325	1.151	1.521	0.701	0.789	-	-	-
	MR438	0.168	0.688	0.874	3.344	0.611	0.663	-	-	-
	T-	1.000	1.000	1.000	1.000	1.000	1.000	-	-	-
D341-MS	tufstine	0.372	0.553	0.762	0.810	1.167	0.960	-	-	-
	MR438	0.493	0.865	0.350	0.922	1.023	0.962	-	-	-

Protein		CD15			CD133			NF-M		
Repition		1	2	3	1	2	3	1	2	3
	T-	1.000	1.000	1.000	-	-	-	1.000	1.000	1.000
**DAOY-MS**	tufstine	0.629	0.964	1.095	-	-	-	1.656	2.846	1.434
	MR438	0.392	0.772	0.855	-	-	-	4.839	9.147	3.203
	T-	1.000	1.000	1.000	1.000	1.000	1.000	1.000	1.000	1.000
**D283-MS**	tufstine	1.039	0.567	0.772	0.693	0.372	0.730	1.096	1.299	2.541
	MR438	0.459	0.880	0.473	0.347	0.779	0.567	2.318	2.369	2.817
	T-	1.000	1.000	1.000	1.000	1.000	1.000	1.000	1.000	1.000
**D341-MS**	tufstine	1.168	0.493	0.840	0.608	0.990	0.319	0.689	0.612	0.904
	MR438	0.810	0.363	0.429	0.781	0.298	0.408	0.521	0.864	0.594

"-": no detectable, $n = 3$.

Supplementary Table 4: Western blot densitometries of peptidomimetic on expression of p-ERK/ERK, p-AKT/AKT, p-SAMAD/β-actin for MB stem cells of MB

Protein		p-ERK/ERK			p-AKT/AKT			p-SAMD		
Repition		1	2	3	1	2	3	1	2	3
DAOY-MS	T-	1.000	1.000	1.000	1.000	1.000	1.000	1.000	1.000	1.000
	tufstine	0.971	0.169	0.270	0.879	0.866	0.735	0.958	0.787	0.744
	MR438	0.827	0.419	0.568	0.792	0.765	0.733	1.417	0.632	0.715
D283-MS	T-	1.000	1.000	1.000	1.000	1.000	1.000	1.000	1.000	1.000
	tufstine	0.856	0.962	0.849	0.369	1.213	0.501	0.724	0.809	0.834
	MR438	0.983	1.082	1.177	0.282	2.015	1.038	1.413	1.477	1.134
D341-MS	T-	1.000	1.000	1.000	1.000	1.000	1.000	1.000	1.000	1.000
	tufstine	1.068	1.360	0.989	0.999	0.494	0.908	1.334	1.122	0.968
	MR438	1.002	1.844	1.094	1.621	0.430	0.282	1.298	1.178	1.422

"-": no detectable, $n = 3$.

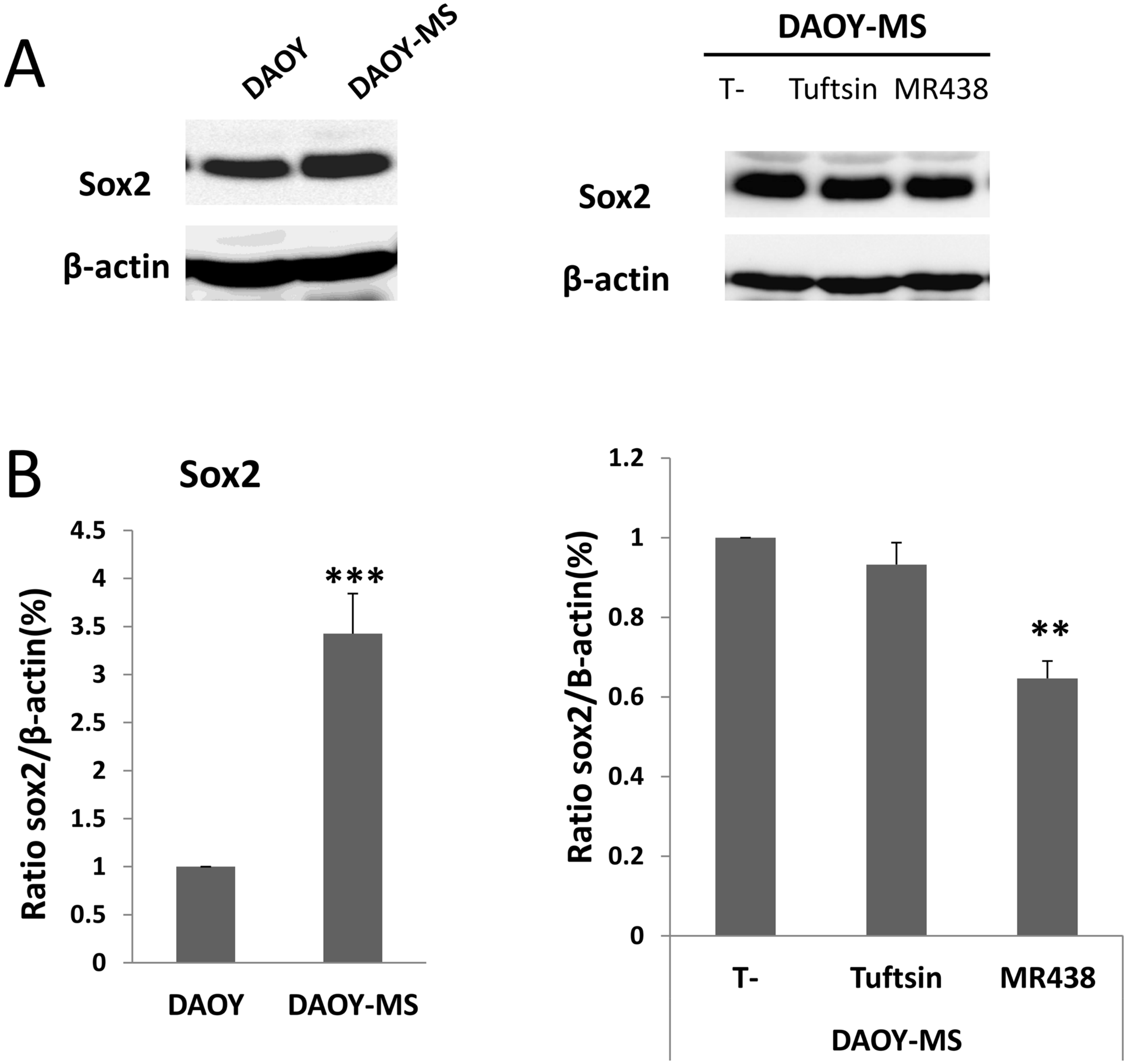

Supplementary Figure 1: Effect of MR438 and Tuftsin on expression of Sox2 for DAOY. (**A**) Representative images of expression of Sox2 for DAOY exposed to MR438 or Tuftsin by Western blot. (**B**) Ratio of Sox2 expression to β-actin protein for MB stem cells treated by MR438 or Tuftsin for DAOY. $^{*}p < 0.05$, $^{**}p < 0.01$, $n = 3$.

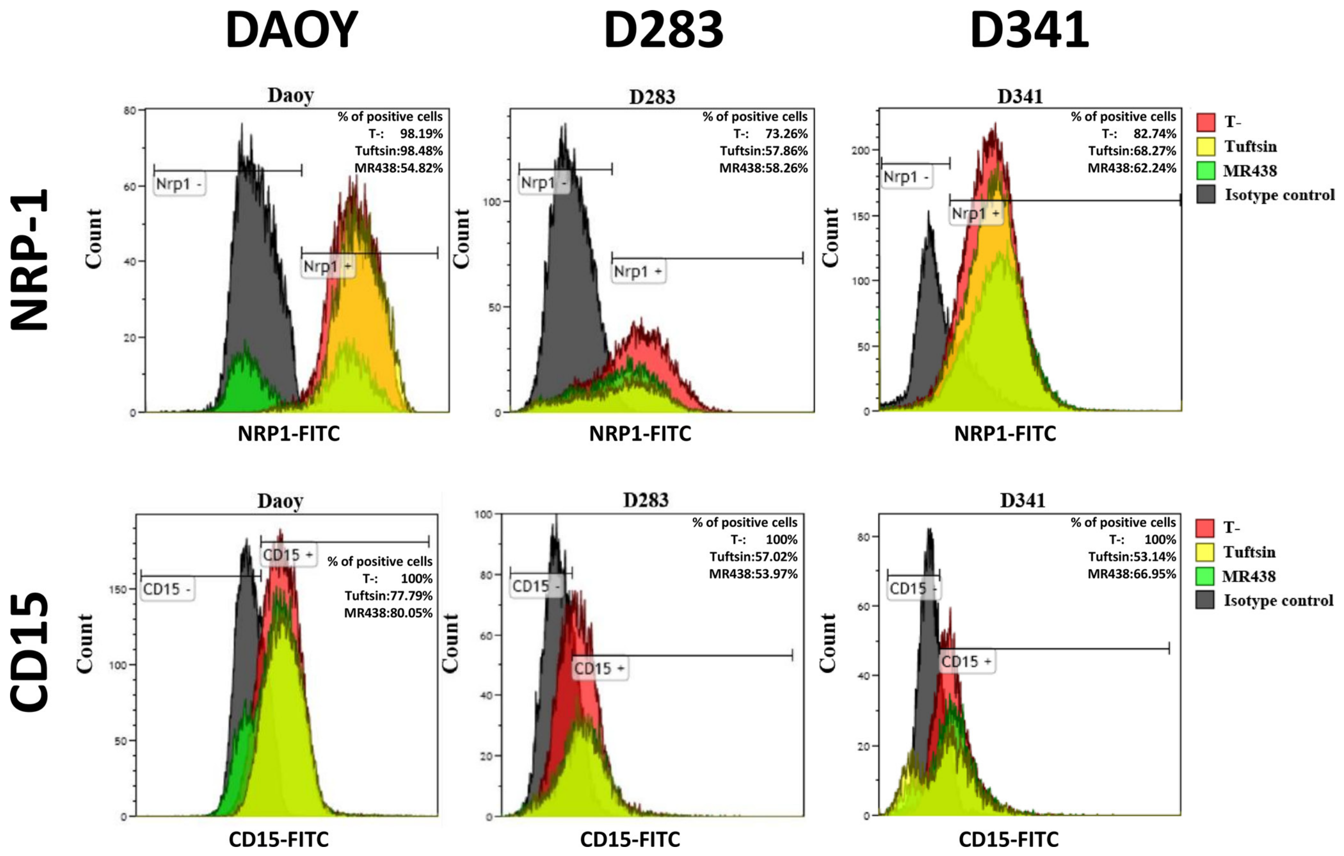

Supplementary Figue 2: Effect of MR438 or Tuftsin on expression of NRP-1 and CD15 by flow cytometry for the 3 MB stem cells models.

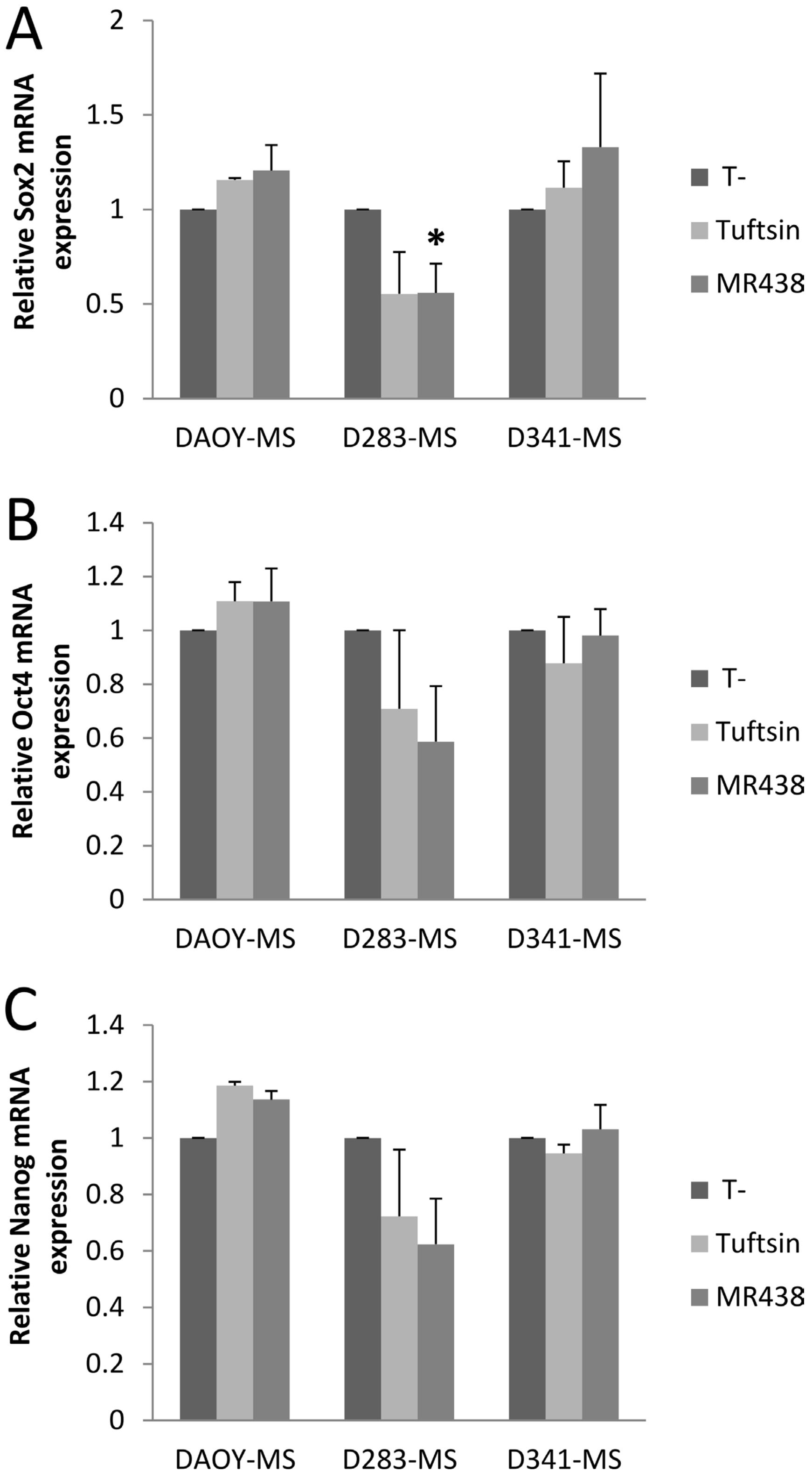

Supplementary Figure 3: Effect of MR438 and Tuftsin on expression of transcripts by qRT-PCR. (**A**) Sox2 (**B**) Oct4 and (**C**) Nanog. $^{*}p < 0.05$, $n = 3$.
